# Broadband Optical Constants and Nonlinear Properties of SnS_2_ and SnSe_2_

**DOI:** 10.3390/nano12010141

**Published:** 2021-12-31

**Authors:** Georgy A. Ermolaev, Dmitry I. Yakubovsky, Marwa A. El-Sayed, Mikhail K. Tatmyshevskiy, Arslan B. Mazitov, Anna A. Popkova, Ilya M. Antropov, Vladimir O. Bessonov, Aleksandr S. Slavich, Gleb I. Tselikov, Ivan A. Kruglov, Sergey M. Novikov, Andrey A. Vyshnevyy, Andrey A. Fedyanin, Aleksey V. Arsenin, Valentyn S. Volkov

**Affiliations:** 1 Center for Photonics and 2D Materials, Moscow Institute of Physics and Technology, 9 Institutsky Lane, 141700 Dolgoprudny, Russia; georgiy.ermolayev@phystech.edu (G.A.E.); dmitrii.yakubovskii@phystech.edu (D.I.Y.); mira@phystech.edu (M.A.E.-S.); mikhail.tatmyshevskiy@phystech.edu (M.K.T.); arslan.mazitov@phystech.edu (A.B.M.); slavich.as@phystech.edu (A.S.S.); celikov@physics.msu.ru (G.I.T.); kruglov.ia@mipt.ru (I.A.K.); novikov.s@mipt.ru (S.M.N.); andrey.vyshnevyy@phystech.edu (A.A.V.); arsenin.av@mipt.ru (A.V.A.); 2 Department of Physics, Faculty of Science, Menoufia University, Shebin El-Koom 32511, Egypt; 3 Dukhov Research Institute of Automatics (VNIIA), 22 Suschevskaya St., 127055 Moscow, Russia; 4 Faculty of Physics, Lomonosov Moscow State University, 119991 Moscow, Russia; popkova@nanolab.phys.msu.ru (A.A.P.); antropov@nanolab.phys.msu.ru (I.M.A.); bessonov@nanolab.phys.msu.ru (V.O.B.); fedyanin@nanolab.phys.msu.ru (A.A.F.)

**Keywords:** two-dimensional materials, optical constants, dielectric properties, refractive index, nanophotonics, spectroscopic ellipsometry, second harmonic generation

## Abstract

SnS_2_ and SnSe_2_ have recently been shown to have a wide range of applications in photonic and optoelectronic devices. However, because of incomplete knowledge about their optical characteristics, the use of SnS_2_ and SnSe_2_ in optical engineering remains challenging. Here, we addressed this problem by establishing SnS_2_ and SnSe_2_ linear and nonlinear optical properties in the broad (300–3300 nm) spectral range. Coupled with the first-principle calculations, our experimental study unveiled the full dielectric tensor of SnS_2_ and SnSe_2_. Furthermore, we established that SnS_2_ is a promising material for visible high refractive index nanophotonics. Meanwhile, SnSe_2_ demonstrates a stronger nonlinear response compared with SnS_2_. Our results create a solid ground for current and next-generation SnS_2_- and SnSe_2_-based devices.

## 1. Introduction

Van der Waals materials have emerged as a promising building block for next-generation optical and electronic devices [1,2,3,4,5,6,7,8]. Their planar structure [9,10] and the outstanding compatibility with existing manufacturing techniques [11,12,13,14,15] make such materials ideal for integration into modern industrial and scientific devices. Among layered materials, graphene [16], MoS_2_ [17], and hBN [18] have received the most attention, as they were the first [19,20,21] to catch researchers’ interest during the “two-dimensional” revolution [22] in material science. However, the number of known layered materials has increased exponentially over the last decade, with more than 1000 layered compounds being isolated and identified [23]. As a result, their properties are largely unexplored, which considerably impedes their application. In particular, the optical properties of tin-based dichalcogenides SnS_2_ and SnSe_2_ [24,25] are mostly unknown, with rare reports [26,27,28,29,30] on their absorption properties. Nonetheless, SnS_2_ and SnSe_2_ have already demonstrated their huge potential in optoelectronic applications, such as field-effect transistors [31,32,33], solar cells [34,35], saturable absorbers [36,37,38], photonic crystals [39,40], and photodetectors [41,42]. Hence, broadband linear and nonlinear optical properties are highly desired for the acceleration of the development of SnS_2_ and SnSe_2_-based devices.

Here, the objective of the present work is the comprehensive optical characterization of SnS_2_ and SnSe_2_. Using spectroscopic ellipsometry and first-principle calculations, we determine the full broadband dielectric tensor of SnS_2_ and SnSe_2_ from ultraviolet to mid-infrared wavelengths (300–3300 nm). The results demonstrate a high dielectric response (*n* > 3) with zero losses in a wide spectral range: 560–3300 nm for SnS_2_ and 1300–3300 nm for SnSe_2_. Moreover, we measured the second-order nonlinear optical susceptibility of SnS_2_ and SnSe_2_ at wavelengths ranging from 750 to 1050 nm. Finally, our results revealed that SnS_2_ is a high refractive index material, which fills the important gap in the visible spectrum between bandgap energies of GaP and TiO_2_, which makes SnS_2_ a promising material for all-dielectric nanophotonics.

## 2. Results and Discussion

### 2.1. Surface and Structural Morphology Study

**Figure 1:**
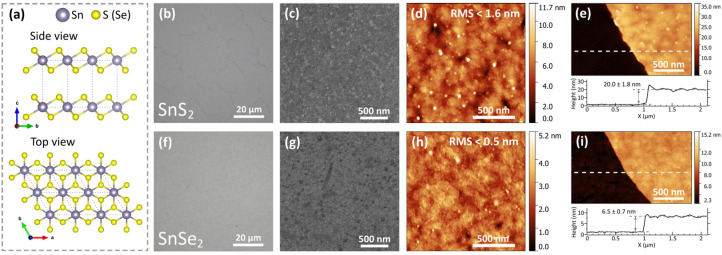
Morphology of SnS_2_ and SnSe_2_. (**a**) Crystal lattice structure of 1T-SnS_2_ (or 1T-SnSe_2_) [44], optical microscopy images of (**b**) SnS_2_ and (**f**) SnSe_2_. SEM images of (**c**) SnS_2_ and (**g**) SnSe_2_. AFM scan images of (**d**) SnS_2_ and (**h**) SnSe_2_. AFM thickness measurements of (**e**) SnS_2_ and (**i**) SnSe_2_ films with characteristic step height profiles.

Thin films of SnS_2_ and SnSe_2_ were synthesized by the chemical vapor deposition (CVD) method and transferred on a quartz substrate. Figure 1a schematically illustrates the crystal structure of 1T-SnS_2_ or SnSe_2_ viewed along *c*- axis and the *a*-axis. This crystal configuration is the most common atoms’ arrangement for SnS_2_ and SnSe_2_, where layers stack directly above one another [43,44]. Optical microscopy photographs in Figure 1b,f show the uniform substrate’s coverage of synthesized SnS_2_ and SnSe_2_ films. Likewise, scanning electron microscopy (SEM) images in Figure 1c,g confirm the films’ full-area coverage and homogeneity at the microscale. In addition, we checked the films’ surface by atomic force microscopy (AFM), demonstrating an atomically smooth surface with root mean square (RMS) roughness of less than 1.6 nm and 0.5 nm for SnS_2_ and SnSe_2_, respectively. Ultimately, we accurately measured the films’ thickness via AFM topographical scans (Figure 1e,i). They yielded 20.0 ± 1.8 nm and 6.5 ± 0.7 nm thicknesses for SnS_2_ and SnSe_2_ films, correspondingly.

### 2.2. Analysis of the Crystal Structure and Raman Characterization

**Figure 2:**
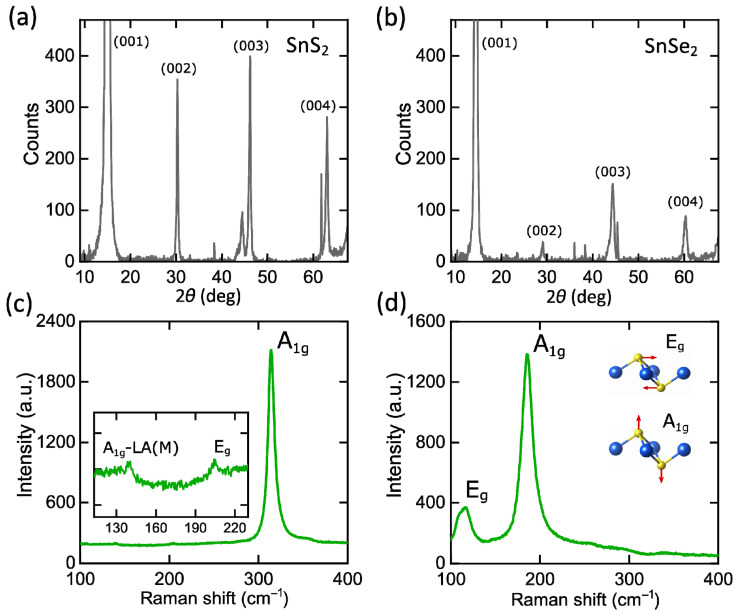
Structural characterization of SnS_2_ and SnSe_2_. XRD patterns of (**a**) SnS_2_ and (**b**) SnSe_2_. Raman spectra for (**c**) SnS_2_ and (**d**) SnSe_2_ thin films.

In nature, SnS_2_ and SnSe_2_ exist in several phase modifications [45,46], including 1T, 2H, 4H, and 18R polytypes. To identify the phase of our samples, we performed X-ray diffraction (XRD), whose spectra are displayed in Figure 2a,b. According to the Joint Committee on Powder Diffraction Standards (card No. 23-0677 and 89-2939) and previous publications [27,47,48], the obtained XRD patterns reveal the hexagonal lattice configuration, which could be 1T or 2H, for SnS_2_ and SnSe_2_ with lattice parameters *a* = *b* = 3.6486 Å and *c* = 5.8992 Å for SnS_2_ and *a* = *b* = 3.811 Å and *c* = 6.137 Å for SnSe_2_.

Aside from XRD characterization, we utilized Raman spectroscopy at 532 nm excitation wavelength (Figure 2c,d) to distinguish between two hexagonal configurations, 1T and 2H. Raman spectrum of SnS_2_ reveals out-of-plane vibration mode A_1g_ at ~314 cm^−1^ and in-plane vibration of E_g_ at ~205 cm^−1^, corresponding to 1T polytype [44,49,50]. Similar to SnS_2_, SnSe_2_ Raman spectrum has two characteristic phonon modes: A_1g_ mode at ~185 cm^−1^ and E_g_ mode at ~116.5 cm^−1^, associated with 1T-phase [36,51]. Moreover, Raman spectra at numerous locations of our samples demonstrate the same A_1g_ and E_g_ peak positions, additionally validating the homogeneity of the studied SnS_2_ and SnSe_2_ thin films.

### 2.3. Optical Properties of SnS_2_ and SnSe_2_ Films

**Figure 3:**
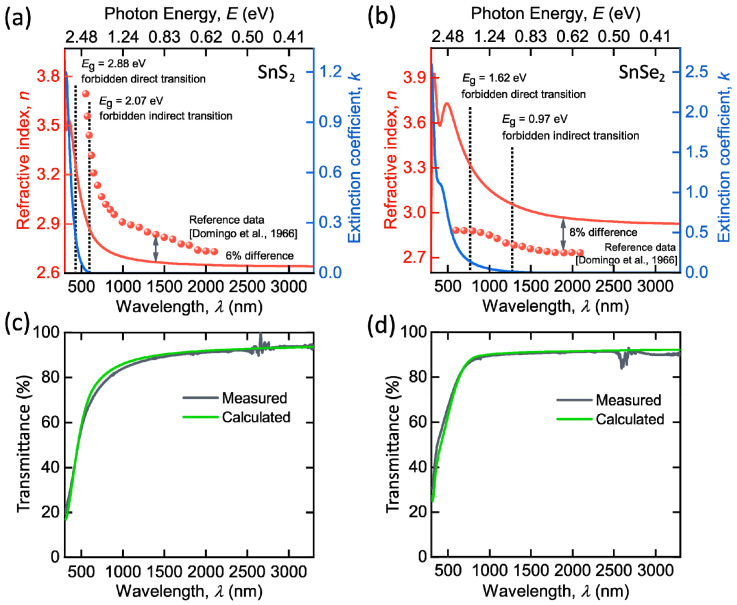
Linear optical properties of SnS_2_ and SnSe_2_**.** Dielectric function of (**a**) SnS_2_ and (**b**) SnSe_2_. For comparison, we included refractive indices (red circles) and electronic transitions (dashed lines) determined by Domingo et al. [26]. Measured and calculated transmittance for (**c**) SnS_2_ and (**d**) SnSe_2_ on quartz. Tabulated optical constants for SnS_2_ and SnSe_2_ are collected in Table A1.

We investigated broadband optical constants of SnS_2_ and SnSe_2_ films through spectroscopic ellipsometry. We employed a two-layer optical model for ellipsometry data analysis: quartz substrate with SnS_2_ or SnSe_2_ film with the thickness determined from AFM (Figure 3e,i). Similar to other TMDCs [52,53], we describe SnS_2_ and SnSe_2_ dielectric function by the Tauc–Lorentz oscillator model (see Methods) [54,55]. Figure 3a,b shows the resulting optical constants *n* and *k* for SnS_2_ and SnSe_2_ films. Interestingly, we did not observe excitons for SnS_2_ and SnSe_2_, which can be explained by their indirect bandgap, in contrast, to the direct bandgap in MoS_2_ and WS_2_ [56,57]. Apart from the dielectric function, Tauc–Lorentz oscillator parameters allow us to obtain the positions of critical points of joint density of states: 3.91 eV (317 nm) for SnS_2_; 2.87 eV (432 nm) and 3.98 eV (311 nm) for SnSe_2_. Furthermore, SnS_2_ and SnSe_2_ both have zero absorption (*k* ~ 0) at a broad wavelength range, starting from 560 and 1300 nm (Figure 3a,b), respectively. For reference, we also plotted in Figure 3a,b refractive indices and bandgap transitions of SnS_2_ and SnSe_2_, determined by Domingo and coworkers [26]. As expected, the fundamental absorption edge coincides with the forbidden indirect transitions (Figure 3a,b), supporting our results in Figure 3a,b. For additional verification, we also measured the transmittance spectra of our samples (Figure 3c,d) and compared them with the transfer matrix calculations [58], based on optical constants from Figure 3a,b. Evidently, calculated and measured transmittance agree well, thereby validating our *n* and *k* in Figure 3a,b.

**Figure 4:**
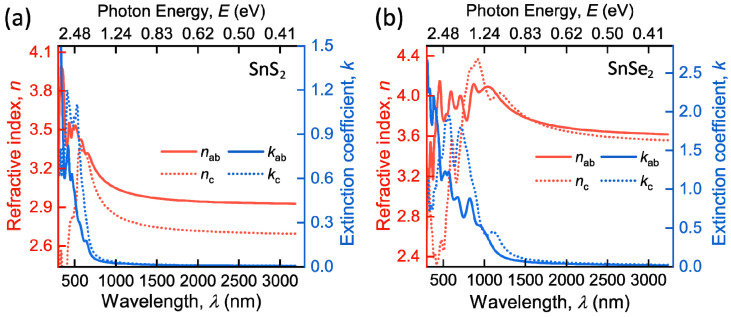
First-principle calculations of SnS_2_ and SnSe_2_**.** Optical constants for (**a**) SnS_2_ and (**b**) SnSe_2_, including in-plane *n*_ab_, *k*_ab_ and out-of-plane *n*_c_, *k*_c_ parts of dielectric tensor.

To retrieve the full dielectric tensor, we leveraged first-principle calculations (Methods). Figure 4 shows the resulting refractive index and extinction coefficient along the *ab*-plane (*n*_ab_ and *k*_ab_) and *c*-axis (*n*_c_ and *k*_c_). The first-principle calculations reproduce the shape of the experimental dielectric function and render the major optical features: a wide zero-absorption spectral range and high dielectric response. However, first-principle calculations overestimate values of dielectric function since the computations were performed assuming the ideal crystalline structure, whereas the studied CVD-grown films have a polycrystalline structure. Nevertheless, first-principle calculations provide access to the full dielectric permittivity tensor, allowing us to estimate the anisotropic optical properties, which are the most noticeable for SnS_2_ with birefringence Δ*n* = *n*_ab_ − *n*_c_ ≈ 0.3 and almost negligible for SnSe_2_. In contrast, ellipsometry is nearly insensitive to optical constants along the *c*-axis, as explained by Ermolaev and colleagues [56,59]. Thus, our computations reveal for the first time the optical anisotropy in SnS_2_ and SnSe_2_, which could be relevant in next-generation anisotropic nanophotonics [60].

**Figure 5:**
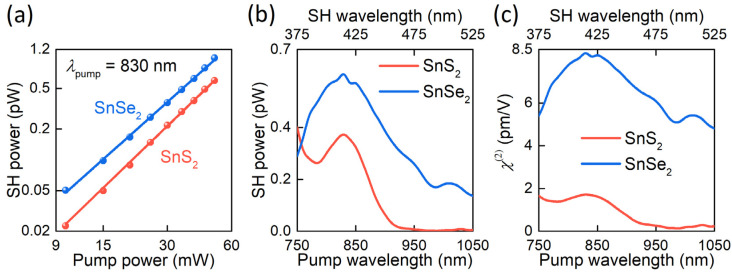
Nonlinear optical properties of SnS_2_ and SnSe_2_. (**a**) Power-dependent nonlinear optical response of SnS_2_ and SnSe_2_ thin films, plotted in double logarithmic scale, and its linear approximation with slope *p* = 2.01 ± 0.02 for SnS_2_ and *p* = 2.02 ± 0.04 for SnSe_2_. Pump wavelength is 830 nm. (**b**) SHG spectroscopy of SnS_2_ (red line) and SnSe_2_ (blue line) thin films at 40 mW pump power. (**c**) Wavelength-dependent, second-order, nonlinear optical susceptibility of SnS_2_ (red line) and SnSe_2_ (blue line).

In the light of the rapid development of nonlinear optical devices based on SnS_2_ and SnSe_2_ [36,37,61], we also measured their nonlinear optical response (Figure 5). Specifically, we measured the second harmonic generation (SHG) in transmission geometry using 150 fs laser pulses focused into a 50 µm spot in diameter (see Methods). Figure 5a shows the SHG power dependence with the expected slope of 2 (2.01 ± 0.02 for SnS_2_ and 2.02 ± 0.04 for SnSe_2_), confirming the second-order nonlinear process and the absence of saturation effects. SHG spectra of SnS_2_ and SnSe_2_ are shown in Figure 5b. For SnSe_2_, SHG resonance is at 415 nm (2.98 eV), associated with the 2 photon direct transition at the critical point (2.87 eV) found above from ellipsometry measurements. The presence of SH signal at large pump wavelengths indicates the contribution of direct transitions with lower energies, meaning that the direct transition of SnSe_2_ is less than 2.36 eV. In contrast, for SnS_2_, the SH signal is negligible at large wavelengths. Therefore, the SHG resonance observed at the SH wavelength of 420 nm (2.95 eV) can be associated with the lowest energy direct transition of SnS_2_ in agreement with Domingo and colleagues’ work [26].

To calculate the nonlinear optical susceptibility, we implemented the method, described in Boyd’s book [62]. The technique relies on the following equation for the average power of SHG transmitted through sample:

(1) $$P\left(2\omega \right)=\frac{16\sqrt{2}|\chi^{\left(2\right)}{|}^{2}\pi SP^{2}\left(\omega \right)L^{2}}{\epsilon_{0}r^{2}f\tau cn_{\omega }^{2}n_{2\omega }\lambda^{2}}{\sin c}^{2}\left(\frac{\Delta kL}{2}\right)$$

where $\chi^{\left(2\right)}$ is a nonlinear optical susceptibility, S = 0.94 is the shape factor for Gaussian pulses, $\epsilon_{0}$ is the permittivity of vacuum, c is the speed of light, f = 80 MHz is the pulse repetition rate, *τ* = 150 fs is the pulse duration, r = 25 µm is the focal spot radius, L is a sample thickness, λ is a pump wavelength, Δk is the wavevectors mismatch of the pump and SH waves, $n_{\omega }$ and $n_{2\omega }$ are refractive indices of material at pump and harmonic wavelengths, and P(ω) and P(2ω) are average power of the pump and the second harmonic radiation, respectively. In our case, the coherence length $L_{coh}=\lambda /\left(4n_{2\omega }-4n_{\omega }\right)$ of the observed processes is several hundred nanometers (from 300 nm to 900 nm for SnS_2_ and from 450 to 600 nm for SnSe_2_), which significantly exceeds the thickness of the films (Figure 1e,f). Thus, we can assume that the SHG is phase-matched and, hence, ${\sin c}^{2}\left(\Delta kL/2\right)=1$. It allows us to evaluate SnS_2_ and SnSe_2_ nonlinear optical susceptibility, displayed in Figure 5c.

**Figure 6:**
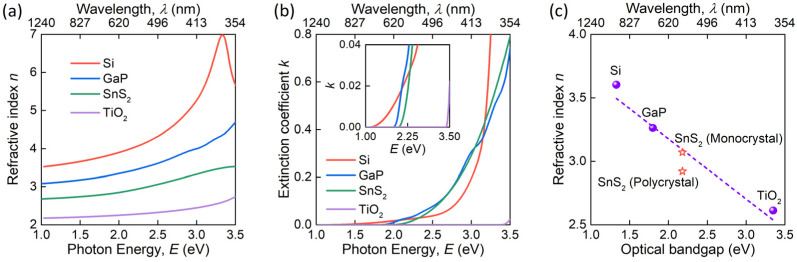
SnS_2_ as a high refractive index material. (**a**) Refractive index *n* and (**b**) extinction coefficient *k* of SnS_2_ compared with other high refractive index materials—Si, GaP, and TiO_2_. (**c**) The dependence of refractive index and optical bandgap for high refractive index materials.

Finally, we want to underline that SnS_2_ is a promising material for all-dielectric nanophotonics [63,64], demanding a high refractive index and low absorption. As shown in Figure 6, SnS_2_ meets both requirements since it possesses a refractive index *n* ≈ 2.8 and zero extinction in the visible and infrared ranges. More importantly, SnS_2_ could even compete with classical high refractive index materials such as Si, GaP, and TiO_2_ [65,66,67,68]. In particular, SnS_2_ has a wider transparency region compared with GaP and Si and a larger refractive index than TiO_2_ (Figure 6). More surprisingly, when we use the refractive index from first-principle calculations (Figure 4a) for monocrystalline SnS_2_, it perfectly fits into the correlation line between the refractive indices and optical bandgaps of high refractive index materials (Figure 6c). Therefore, SnS_2_ enables the essential spectral range of all-dielectric nanophotonics between GaP and TiO_2_.

## 3. Materials and Methods

### 3.1. Materials

CVD-grown full-area coverage SnS_2_ and SnSe_2_ samples of thin films were purchased from 2d Semiconductors Inc. (2d Semiconductors Inc., Scottsdale, AZ, USA). The samples with an area of 1 × 1 cm^2^ were grown by CVD on sapphire substrates and subsequently transferred on quartz substrates.

### 3.2. Surface Morphology Characterization

The surface morphology of SnS_2_ and SnSe_2_ thin films was analysed by an optical microscope (Nikon LV150, Tokyo, Japan) with a digital camera DS-Fi3, as well as the scanning electron microscope (SEM) using the acceleration voltage of 30 kV and different magnifications (JEOL JSM-7001F, Tokyo, Japan) to prove films homogeneity. The film surface morphology was studied by atomic force microscopy (AFM, notegra, Nt-MDT Spectrum Instruments, Moscow, Russia) in semi-contact mode using a silicon tip with a radius <10 nm and resonance frequency of ~250 kHz (HA_NC Etalon, Tipsnano, Tallinn, Estonia) to determine surface roughnesses and films thicknesses.

### 3.3. Crystal Structure Characterization

X-ray diffraction (XRD) characterization was performed by X-ray diffractometer (ARL X’TRA, Thermo Fisher Scientific, Waltham, MA, USA) using Cu K_α1_ radiation line (λ = 1.54 Å) to analyze the crystal structure of the films using a regime of 2*θ*-scan with angles range of 5°–75° with a step of 0.05° and accumulation time of 2 s.

### 3.4. Raman Characterization

The Raman spectra were measured with a confocal scanning Raman microscope Horiba LabRAM HR Evolution (HORIBA Ltd., Kyoto, Japan) with 532 nm linearly polarized excitation laser, 1800 lines/mm diffraction grating, and ×100 objective (N.A. = 0.90) using a spectra range of 100–450 cm^−1^. The spectra were recorded with 3.5 mW incident laser power, with an integration time of 10 s and 10 spectra accumulation.

### 3.5. Ellipsometry Analysis

The optical constants *n* and *k* of SnS_2_ and SnSe_2_ were measured using a variable-angle spectroscopic ellipsometer (VASE, J.A. Woollam Co., Lincoln, NE, USA), working at room temperature, at variable incidence angles 30°–75° with a step of 5° and wide spectral range from 300 to 3300 nm with a step of 1 nm, having the spotlight of size ~1 mm around the center of the sample, utilizing the high precision optical alignment. To fit the measured ellipsometric parameters *Ψ* and *Δ*, we used the Tauc–Lorentz oscillator model was used, defined by the following formula:

(2) $$\varepsilon_{2}=\{\begin{array}{c} \frac{1}{E}\cdot \frac{AE_{0}C{\left(E-E_{g}\right)}^{2}}{{\left(E^{2}-E_{0}^{2}\right)}^{2}+C^{2}E^{2}}\;for\;E>E_{g}\\ 0\;\;\;\;\;\;\;\;\;\;\;\;\;\;\;\;\;\;\;\;\;\;\;\;\;\;\;\;\;for\;E<E_{g} \end{array},$$

where E is the energy of the photon, A is the oscillator strength, C is the oscillator broadening, $E_{g}$ is the optical band-gap, $E_{0}$ is the oscillator central energy, and the real part of the dielectric function $\varepsilon_{1}$ was obtained from the imaginary part $\varepsilon_{2}$ using the Kramers–Kronig integration, plus $\varepsilon_{\infty }$, to account for high energy electronic transitions. For SnS_2_, we used one Tauc–Lorentz oscillator with the following parameters: A= 54.613 eV; C= 1.626 eV; $E_{0}=$ 3.911 eV; $E_{g}=$ 1.970 eV and $\varepsilon_{\infty }=$ 5.031. For SnSe_2_, we used two Tauc–Lorentz oscillators with the following parameters: $A_{1}=$ 14.435 eV; $C_{1}=$ 1.345 eV; $E_{01}=$ 2.870 eV; $A_{2}=$ 20.432 eV; $C_{2}=$ 0.875 eV; $E_{02}=$ 3.981 eV; $E_{g}=$ 0.736 eV and $\varepsilon_{\infty }=$ 4.445.

### 3.6. Optical Properties Characterization

Optical transmittance spectra of SnS_2_ and SnSe_2_ films on quartz were measured with a spectrophotometer (Cary 5000 UV-Vis-NIR, Agilent Tech., Santa Clara, CA, USA) at a wavelength range of 300–3300 nm.

The nonlinear optical properties of the sample were studied by a home-built multiphoton microscope [69], based on femtosecond Ti:sapphire laser (Coherent Chameleon Ultra 2, Santa Clara city, CA, USA) tunable in the spectral range from 680 to 1080 nm. The laser beam (80 MHz repetition rate, 150 fs pulse duration) was directed through the system, consisting of a half-wave plate on a motorized rotation stage and a Glan–Taylor prism, which provided control of the power and polarization of the incident radiation.

Then, the beam was focused on the sample surface with a 10 cm lens into a 50 μm spot. The sample was mounted on a 3-axis motorized stage (SigmaKoki, Tokyo, Japan) with a minimum step of 0.1 μm, which made it possible to accurately align the sample relative to the pump spot. The SH radiation generated by the sample was collected by an objective lens (N.A. = 0.95, 100x, Olympus, Tokyo, Japan) and directed to the detection channel consisting of a tube lens, filter (FGB39 Thorlabs, Newton, NJ, USA) to cut off the pump radiation, monochoromator, and a scientific CCD camera (Andor Clara, Belfast, United Kingdom). The SH signal was normalized over spectral functions of all optical elements in the detection channel including objective lens transmittance and detector sensitivity spectra. SHG spectra were measured at the same pump intensity for all wavelengths. The experimental setup was fully automated and situated in a black box.

### 3.7. First-Principle Calculations

The optical properties of SnS_2_ and SnSe_2_ were calculated using density functional theory (DFT) implemented in the Vienna Ab Initio Simulation Package [70,71]. Core electrons, their interaction with valence electrons, and exchange correlation effects were described within generalized gradient approximation [72] (Perdew–Burke–Ernzerhof functional) and the projector-augmented wave pseudopotentials [73]. The unit cell parameters were *a* = *b* = 3.6486 Å and *c* = 5.8992 Å for SnS_2_ and *a* = *b* = 3.811 Å and *c* = 6.137 Å for SnSe_2_. The calculation was performed in two steps: first, the atomic positions of SnS_2_ and SnSe_2_ were relaxed in until the interatomic forces were less than 10^−3^ eV/Å, and a 1-electron basis set was obtained from a standard DFT calculations. Second, the real and imaginary parts of frequency-dependent dielectric function were calculated using the GW approximation [74]. Cutoff energy of the plane waves basis set was set to 600 eV, and the Γ-centered 11 × 11 × 7 k-points mesh was used to sample the first Brillouin zone.

## 4. Conclusions

In conclusion, we theoretically and experimentally determined the anisotropic optical constants of SnS_2_ and SnSe_2_ in a wide spectral range (300–3300 nm). Our findings reveal a strong dielectric response of SnS_2_ and SnSe_2_ and their broad range with zero absorption. More importantly, for SnS_2_, this range includes visible frequencies, which makes SnS_2_ a novel high refractive index material, which complements the classical high refractive index materials Si, GaP, and TiO_2_. Additionally, we measured the second-order nonlinear susceptibility of SnS_2_ and SnSe_2_. From a broader perspective, our research enables a foundation for advanced optical engineering with SnS_2_ and SnSe_2_.

## Data Availability

The data presented in this study are available upon reasonable request from the corresponding author.

## Appendix A

**Table A1:** Tabulated optical constants for SnS_2_ and SnSe_2_ films from Figure 3a,b.

	SnS_2_		SnSe_2_	
*λ* (nm)	*n*	*k*	*n*	*K*
300	3.8943	1.0436	2.8895	2.5984
350	3.5319	0.8434	3.8561	1.3915
400	3.3828	0.3664	3.5830	1.1143
450	3.1896	0.1537	3.6856	0.9836
500	3.0450	0.0599	3.7271	0.7105
550	2.9415	0.0180	3.6563	0.4900
600	2.8674	0.0021	3.5609	0.3480
650	2.8171	0.0000	3.4737	0.2566
700	2.7841	0.0000	3.4004	0.1950
750	2.7602	0.0000	3.3399	0.1515
800	2.7420	0.0000	3.2897	0.1195
850	2.7277	0.0000	3.2477	0.0952
900	2.7163	0.0000	3.2122	0.0762
1200	2.6782	0.0000	3.0787	0.0190
1500	2.6621	0.0000	3.0115	0.0021
1800	2.6537	0.0000	2.9751	0.0000
2100	2.6488	0.0000	3.6446	0.0000
2400	2.6456	0.0000	3.6001	0.0000
2700	2.6434	0.0000	3.5663	0.0000
3000	2.6419	0.0000	3.5400	0.0000
3300	2.6408	0.0000	3.5194	0.0000
